# The Role of Plant-Based Nutrition and Exercise in Metabolic Syndrome: A Narrative Review

**DOI:** 10.3390/nu17091498

**Published:** 2025-04-29

**Authors:** James Stavitz, Ryan Porcelli, Jennifer Gentile

**Affiliations:** 1 Department of Athletic Training Education, Kean University, Union, NJ 07083, USA; 2 Department of Physical Therapy, Kean University, Union, NJ 07083, USA

**Keywords:** metabolic syndrome, plant-based nutrition, exercise intervention, insulin sensitivity, cardiovascular health

## Abstract

Background/Objectives: Metabolic syndrome (MetS) is a prevalent health condition characterized by central obesity, insulin resistance, hypertension, and dyslipidemia, increasing the risk of cardiovascular disease and type 2 diabetes. Lifestyle interventions, particularly plant-based nutrition and exercise, are essential for managing MetS. While both strategies are well-documented independently, their synergistic effects remain less explored. This narrative review integrates findings from both domains to evaluate their combined impact on metabolic syndrome. The review examines the individual and combined impacts of plant-based nutrition and exercise on MetS-related metabolic dysfunction. Methods: A comprehensive review of 114 peer-reviewed studies was conducted to assess the role of plant-based diets and structured physical activity in improving insulin sensitivity, lipid profiles, inflammation, and weight management. Studies investigating the mechanisms through which dietary components and exercise modalities influence metabolic health were analyzed, along with behavioral and psychological factors affecting long-term adherence. Results: Plant-based diets, particularly those high in fiber, polyphenols, and healthy fats, improve glucose metabolism, reduce inflammation, and enhance cardiovascular health. Exercise complements these benefits by increasing insulin sensitivity, promoting fat oxidation, and improving lipid metabolism. When combined, plant-based nutrition and exercise provide superior metabolic outcomes, including greater reductions in visceral adiposity, improved endothelial function, and enhanced glycemic control. Conclusions: Plant-based nutrition and structured exercise are effective strategies for managing MetS. Their synergistic effects highlight the importance of integrated lifestyle interventions for long-term metabolic health.

## 1. Introduction

Metabolic syndrome (MetS) is a growing public health issue that significantly increases the risk of cardiovascular disease, type 2 diabetes, and other chronic conditions [1]. It is defined by a combination of central obesity, insulin resistance, high blood pressure, and abnormal lipid levels, all of which contribute to poor metabolic health [2]. Over the past few decades, the prevalence of MetS has risen dramatically, largely due to poor dietary habits, sedentary lifestyles, and the increasing rates of obesity [3]. As these risk factors continue to rise, addressing them has become a priority in reducing the long-term health consequences of MetS.

One of the most effective ways to manage metabolic syndrome is through lifestyle interventions, particularly nutrition and physical activity [4,5]. Plant-based diets, which emphasize vegetables, fruits, legumes, whole grains, nuts, and seeds, have gained increasing attention for their metabolic benefits [5,6]. These diets are naturally low in energy density, averaging approximately 0.5 to 1.5 kcal per gram compared to 2.5 to 4.0 kcal per gram in more energy-dense Western dietary patterns [6]. This lower calorie density allows individuals to consume satisfying portions while reducing total caloric intake, supporting weight loss and improved insulin sensitivity [6,7]. Plant-based diets are also high in fiber, antioxidants, and healthy fats, all of which contribute to improved glycemic control, lipid profiles, and inflammation reduction [6,7,8].

While the benefits of plant-based diets and exercise are well-documented separately, there is less research on how they work together to influence metabolic syndrome [9]. Understanding their combined effects could lead to better public health recommendations and clinical strategies for managing this condition. While many existing reviews focus on either plant-based nutrition or structured exercise, few synthesize their overlapping mechanisms and combined outcomes. This review fills that gap by examining how these two lifestyle interventions, when implemented together, can produce additive or synergistic benefits for managing metabolic syndrome. This narrative review explores the current evidence on the impact of plant-based nutrition and exercise on metabolic health, focusing on their individual and synergistic effects in reducing the risk factors associated with MetS.

It is important to clarify that “plant-based nutrition” in this review refers to dietary patterns that prioritize whole, minimally processed plant foods. These include vegetables, fruits, legumes, nuts, seeds, and whole grains. Plant-based diets may reduce or eliminate the intake of animal products. Vegan diets, which completely exclude all animal-derived foods, represent the most restrictive form of plant-based eating. This term does not exclusively refer to veganism. Rather, plant-based diets exist on a spectrum, including vegan, vegetarian, Mediterranean, and flexitarian approaches, all of which can be tailored to individual preferences and health needs.

## 2. Materials and Methods

This review focused on studies examining the effects of plant-based nutrition and exercise interventions on MetS. To identify relevant articles, we searched the PubMed, Google Scholar, Scopus, Web of Science, and Cochrane Library databases using the following search terms: “metabolic syndrome”, “insulin resistance”, “cardiovascular disease”, “plant-based diet”, “vegan diet”, “vegetarian diet”, “Mediterranean diet”, “exercise”, “physical activity”, “resistance training”, and “high-intensity interval training (HIIT)”. Only peer-reviewed observational studies, randomized controlled trials (RCTs), clinical trials, systematic reviews, and meta-analyses published in English were included. Studies were considered eligible if they investigated the impact of plant-based diets or specific exercise interventions on metabolic health outcomes. These outcomes included insulin sensitivity, lipid profiles, inflammation, body composition, and cardiovascular health. Eligible studies focused on adults with metabolic syndrome or related metabolic risk factors such as obesity, hypertension, or dyslipidemia. Studies focusing solely on pharmacological treatments, animal models, case reports, editorials, or those lacking metabolic health outcomes were excluded. After screening and eligibility assessment, a total of 114 peer-reviewed articles were included in this review.

## 3. Plant-Based Nutrition and Metabolic Syndrome

Diet plays a fundamental role in both the development and management of metabolic syndrome, with numerous dietary patterns, including plant-based, vegetarian, Mediterranean, and flexitarian diets, showing positive metabolic effects [10]. Research has increasingly shown that plant-based diets, which focus on whole grains, legumes, fruits, vegetables, nuts, and seeds, while limiting or eliminating animal products and processed foods, offer significant metabolic health benefits (Table 1) [11,12,13]. These diets are naturally high in fiber, antioxidants, and unsaturated fats, while being low in saturated fat, cholesterol, and refined sugars, making them an effective strategy for addressing the key metabolic imbalances associated with MetS (Table 2) [12,14].

Specific vegetables and plant components vary in their nutrient density and metabolic effects. Leafy greens like kale, spinach, and Swiss chard are rich in magnesium and nitrates, which help regulate blood pressure and support endothelial function [2]. Cruciferous vegetables such as broccoli, Brussel sprouts, and cauliflower contain glucosinolates and polyphenols, which have demonstrated antioxidant and anti-inflammatory effects [5]. Root vegetables like carrots and beets provide dietary fiber and beta-carotene. Legumes, including lentils, chickpeas, and black beans, are high in plant-based protein, resistant starch, and soluble fibers that benefit glycemic control and lipid profiles [5,6]. These examples illustrate how various anatomical parts of plants, leaves, roots, seeds, and stems offer different bioactive compounds that contribute to metabolic health.

One of the primary ways plant-based nutrition supports metabolic health is by improving insulin sensitivity [15]. High-fiber foods slow glucose absorption, helping to stabilize blood sugar levels and reduce insulin resistance [15,22]. Moreover, plant-derived polyphenols and phytochemicals, found in foods like berries, green tea, and dark leafy greens, have been shown to have anti-inflammatory and antioxidant effects, which help protect against oxidative stress and chronic inflammation—two major drivers of metabolic dysfunction [15,22,23].

Cardiovascular health is another area where plant-based diets provide significant benefits [24]. Unsaturated fats from sources like nuts, seeds, and olive oil help lower LDL cholesterol and triglycerides, while fiber-rich foods like legumes and whole grains can further support healthy lipid metabolism by promoting cholesterol excretion [25]. As a result, plant-based dietary patterns are associated with a lower risk of hypertension, heart disease, and other cardiovascular complications commonly linked to MetS [18,25].

When comparing vegan and vegetarian diets, evidence suggests that vegan diets may provide stronger improvements in certain metabolic parameters due to the complete elimination of animal products. A review by Banaszak et al. (2022) reported that vegan diets are associated with greater reductions in LDL cholesterol (up to 15–25%) compared to vegetarian diets (typically 8–12%) [15]. Additionally, vegan diets tend to result in larger decreases in body weight and fasting glucose levels due to their lower energy density and higher fiber content. In contrast, vegetarian diets, which often include eggs and dairy, may provide more moderate metabolic benefits, but are sometimes easier to adhere to long-term. A meta-analysis by Yokoyama et al. (2014) found that both vegan and vegetarian diets significantly improved glycemic control in type 2 diabetes patients, but the effect size was greater in vegan interventions (HbA1c reduction of −0.4%) compared to vegetarian ones (−0.2%) [18,25,26]. These findings highlight the potential for both dietary approaches to improve metabolic outcomes, while suggesting that stricter plant-based diets may offer greater benefits in certain areas.

### 3.1. Impact on Insulin Sensitivity and Glucose Regulation

Insulin resistance is one of the main drivers of metabolic syndrome and a significant risk factor for type 2 diabetes [26]. It occurs when cells become less responsive to insulin, causing blood sugar levels to rise and forcing the pancreas to produce even more insulin to compensate [27]. Over time, this leads to beta-cell dysfunction, chronic hyperglycemia, and increased metabolic stress [26,27,28]. Plant-based diets have been shown to improve insulin sensitivity through multiple mechanisms, largely due to their high fiber content, low glycemic load, and abundance of bioactive compounds [28,29].

One of the primary ways plant-based diets enhance insulin function is through their high fiber content [15]. Soluble fiber, found in whole grains, legumes, and non-starchy vegetables, slows glucose absorption in the gut, preventing rapid blood sugar spikes after meals [22]. This helps maintain stable glucose levels and reduces the body’s demand for insulin [30]. Many plant-based foods also have a low glycemic index (GI), meaning they cause a slower and more gradual rise in blood sugar, which supports long-term glycemic control and insulin efficiency [29,30,31].

**Figure 1:**
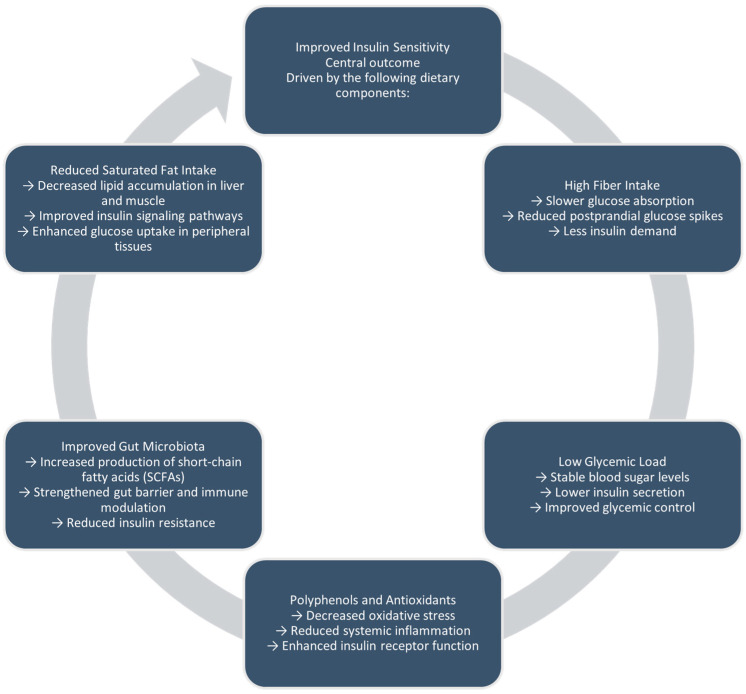
Proposed mechanisms linking plant-based diets to improved insulin sensitivity and glucose regulation.

Plant-based diets boost insulin sensitivity through multiple mechanisms. High fiber intake slows glucose absorption and increases satiety, while a low glycemic load helps reduce blood sugar spikes [29]. Additionally, bioactive compounds such as polyphenols reduce oxidative stress and inflammation. Also, improvements in gut microbiota composition lead to greater production of short-chain fatty acids, which help sustain intestinal barrier function and control immune responses [27,28,29]. Collectively, these effects support more efficient insulin signaling and glucose use in peripheral tissues (Figure 1).

In addition to fiber, polyphenols, which are bioactive compounds abundant in plant foods such as berries, green tea, and dark leafy greens, play a crucial role in improving insulin signaling [32]. These compounds, including flavonoids and catechins, help reduce oxidative stress and inflammation, both of which contribute to insulin resistance [33]. In addition, polyphenols have been shown to stimulate GLUT4 translocation, a process that enables muscle cells to take up glucose more efficiently, improving insulin-dependent glucose metabolism [32,33,34].

### 3.2. Effects on Lipid Profiles and Cardiovascular Health

Dyslipidemia, a condition characterized by elevated triglycerides, low HDL cholesterol, and high LDL cholesterol, is a major risk factor for cardiovascular disease and a key component of metabolic syndrome [35]. Unhealthy lipid profiles contribute to atherosclerosis, hypertension, and increased cardiovascular stress, making lipid regulation essential for metabolic health [35,36]. Research indicates that plant-based diets play a significant role in improving lipid metabolism, reducing the burden of dyslipidemia, and supporting overall cardiovascular function [16,35,36].

One of the primary reasons plant-based diets are effective in improving lipid profiles is their low saturated fat and cholesterol content [17]. Vegan diets, which exclude all animal products, provide zero dietary cholesterol, as cholesterol is only found in animal-derived foods [37]. In contrast, lacto-ovo vegetarian diets may contain moderate levels of dietary cholesterol, typically ranging from 100 to 200 mg per day, depending on the inclusion of eggs and dairy products. Standard Western diets often exceed 300 mg per day in cholesterol intake [37]. Lower dietary cholesterol intake has been consistently associated with reduced LDL cholesterol levels, making vegan diets particularly effective in supporting cardiovascular health [17,37]. To add, plant-based diets replace saturated fats with unsaturated fats from sources like nuts, seeds, avocados, and olive oil, which help increase HDL cholesterol and improve lipid balance [20,37].

Furthermore, plant-based diets contain plant sterols, natural compounds found in legumes, whole grains, and vegetable oils, which compete with dietary cholesterol for absorption in the intestines [38]. This reduces total cholesterol levels and prevents excessive LDL accumulation in the bloodstream. Another key component of plant-based nutrition is soluble fiber, abundant in foods like oats, flaxseeds, and legumes. Soluble fiber binds to cholesterol in the digestive tract and promotes its excretion from the body, further lowering LDL cholesterol and overall cardiovascular risk [39].

Beyond cholesterol regulation, plant-based foods also contain anti-inflammatory and antioxidant compounds that reduce oxidative stress and arterial inflammation, further lowering the risk of atherosclerosis and hypertension [14]. This combination of fiber, healthy fats, plant sterols, and antioxidants makes plant-based diets a powerful tool in protecting cardiovascular health and managing metabolic syndrome.

### 3.3. Role in Inflammation and Oxidative Stress

Chronic low-grade inflammation is a central feature of metabolic syndrome, and contributes to insulin resistance, vascular dysfunction, and dyslipidemia [40,41]. Persistent inflammation alters normal metabolic signaling pathways and increases oxidative stress, which accelerates endothelial damage and metabolic dysfunction [42,43,44]. A major contributor to this cycle is the Western diet, which is high in saturated fat, refined carbohydrates, and processed meat, all of which stimulate pro-inflammatory responses and promote excess reactive oxygen species (ROS) production [17,37]. In contrast, plant-based diets are naturally rich in anti-inflammatory and antioxidant compounds that help restore metabolic balance.

Specific polyphenols, such as quercetin (in apples and onions), catechins (in green tea), and resveratrol (in grapes), play a role in modulating inflammatory signaling pathways. They do so by inhibiting NF-κB activation and reducing the expression of pro-inflammatory cytokines like IL-6 and TNF-α [19,32,33]. These compounds also boost the activity of endogenous antioxidant enzymes such as superoxide dismutase (SOD) and glutathione peroxidase, which help neutralize ROS and protect against oxidative damage [42,45]. In addition, the high fiber content in plant-based diets promotes a diverse gut microbiota that produces short-chain fatty acids (SCFAs), such as butyrate and propionate, which improve gut barrier integrity and suppress systemic inflammation [21,45,46]. These integrated effects contribute to lower levels of circulating inflammatory markers, including C-reactive protein (CRP), and enhance metabolic resilience [45].

Research has linked plant-based diets to significantly lower levels of C-reactive protein (CRP), a key inflammatory marker associated with metabolic syndrome and cardiovascular disease risk [21]. Similarly, the high fiber content in plant-based diets plays a crucial role in supporting gut microbiome diversity, which has been found to regulate immune responses and decrease inflammation [46]. A well-balanced gut microbiome produces short-chain fatty acids (SCFAs) that help maintain gut barrier integrity, preventing harmful compounds from triggering systemic inflammation [21,45,46].

### 3.4. Weight Management and Satiety

Obesity, particularly central adiposity (excess fat around the abdomen), is a major risk factor for metabolic syndrome [47]. Excess visceral fat contributes to insulin resistance, chronic inflammation, and cardiovascular dysfunction, all of which drive metabolic disease progression. Because of this, effective weight management is essential for improving metabolic health [48]. Plant-based diets offer a sustainable and natural approach to achieving and maintaining a healthy weight, making them an important tool for metabolic syndrome prevention and treatment [47,48,49].

One of the key reasons plant-based diets support weight management is their high fiber and water content, which promotes satiety and appetite regulation. Unlike processed, calorie-dense foods that are quickly digested, fiber-rich plant foods slow gastric emptying, keeping individuals fuller for longer [50]. Dietary fiber also plays a role in hormonal regulation of hunger, stimulating the release of peptide YY (PYY) and glucagon-like peptide-1 (GLP-1), two satiety hormones that help reduce appetite and promote fullness. This natural appetite control mechanism allows individuals to consume fewer calories without the need for restrictive dieting, making plant-based nutrition an effective strategy for long-term weight maintenance [50,51].

Research consistently shows that individuals following plant-based diets tend to have lower body mass index (BMI) and reduced prevalence of obesity compared to those consuming omnivorous diets [7,21]. A cross-sectional analysis of over 60,000 adults in the Adventist Health Study-2 found that vegans had the lowest average BMI (23.6 kg/m^2^), followed by vegetarians (25.7 kg/m^2^), pescatarians (26.3 kg/m^2^), and nonvegetarians (28.8 kg/m^2^) [52]. Similarly, a randomized controlled trial demonstrated that overweight participants who adopted a low-fat vegan diet for 16 weeks lost significantly more weight (an average of 6.5 kg) compared to the control group following a standard diet [21]. These outcomes are likely attributed to the higher fiber and water content, lower energy density, and improved satiety associated with plant-based eating patterns. In particular, reductions in visceral adiposity have been observed among plant-based dieters, which is a critical factor in improving insulin sensitivity and reducing metabolic risk [52].

It is also important to consider food preparation methods, which can influence nutrient bioavailability and metabolic outcomes. Steaming vegetables tends to preserve polyphenols and antioxidant capacity, while boiling may reduce water-soluble vitamins like vitamin C and some B-complex nutrients [7,9]. Roasting can improve taste and texture, enhancing adherence, but may slightly reduce certain phytochemicals. Smoking or deep-frying, however, can introduce harmful compounds such as polycyclic aromatic hydrocarbons (PAHs) and trans fats, which negatively impact cardiovascular health [17]. These considerations are especially relevant for individuals with gastrointestinal disorders, such as small intestinal bacterial overgrowth (SIBO), who may benefit from limiting high-FODMAP vegetables (e.g., onions, garlic, Brussels sprouts) and instead opting for low-FODMAP options such as zucchini, eggplant, carrots, and spinach [40]. Tailoring plant-based dietary patterns to individual tolerances enhances both effectiveness and sustainability.

While high-fiber diets are associated with improved satiety and metabolic outcomes, it is important to consider hydration status when increasing fiber intake [22]. Soluble and insoluble fibers interact differently with water in the digestive tract, and adequate fluid intake can help prevent gastrointestinal discomfort such as bloating or constipation in some individuals [39]. However, hydration needs may vary depending on individual tolerance, baseline water consumption, and the type of fiber consumed. Gradual adjustments to fiber intake, alongside attention to hydration, can help maximize the metabolic benefits of fiber without adverse effects [51].

## 4. Exercise and Metabolic Syndrome

Regular physical activity plays a critical role in preventing and managing metabolic syndrome, offering multiple benefits for metabolic and cardiovascular health. Exercise helps improve insulin sensitivity, reduce body fat, optimize lipid metabolism, and support cardiovascular function, making it one of the most effective lifestyle interventions for individuals with metabolic syndrome [53]. By promoting better glucose regulation, improved body composition, and enhanced cardiovascular efficiency, exercise helps address the key risk factors that drive metabolic dysfunction [53,54].

Different types of exercise provide distinct metabolic benefits, and incorporating a variety of training methods can yield the best results (Table 3). Aerobic exercise, such as walking, running, cycling, or swimming, strengthens the cardiovascular system, improves glucose metabolism, and promotes fat oxidation, all of which are crucial for individuals with metabolic syndrome [55]. Resistance training, including weightlifting and bodyweight exercises, helps increase lean muscle mass which, in turn, boosts resting metabolic rate and improves insulin function by allowing muscle cells to take up glucose more efficiently [55,56]. To add, high-intensity interval training (HIIT) has gained attention as a time-efficient and highly effective strategy for metabolic health. HIIT involves short bursts of intense effort followed by brief recovery periods, making it particularly beneficial for reducing visceral fat, rapidly improving cardiovascular fitness, and enhancing insulin sensitivity in a shorter amount of time compared to traditional endurance training [55,56,57].

### 4.1. Effects on Insulin Sensitivity and Glucose Metabolism

Exercise is one of the most effective lifestyle interventions for enhancing insulin sensitivity and improving glucose metabolism, both of which are critical for individuals with metabolic syndrome [58]. Insulin resistance, a hallmark of metabolic syndrome, occurs when cells become less responsive to insulin, leading to elevated blood sugar levels and increased insulin production by the pancreas [26]. Over time, this excess demand on the pancreas can contribute to beta-cell dysfunction, worsening glucose regulation, and increasing the risk of type 2 diabetes [59]. Exercise directly counteracts these effects by enhancing glucose uptake, improving insulin signaling, and optimizing energy metabolism in skeletal muscle [59,60].

One of the primary mechanisms through which exercise improves insulin sensitivity is by stimulating glucose transporter type 4 (GLUT4) translocation. GLUT4 is responsible for facilitating glucose entry into muscle cells, and its activation is typically insulin-dependent. However, during physical activity, muscle contractions independently trigger GLUT4 translocation, allowing glucose to enter cells without the need for insulin. This bypasses insulin resistance and lowers circulating blood sugar levels, reducing the strain on pancreatic beta cells [61].

Both aerobic exercise and resistance training contribute significantly to improving glucose metabolism, but they do so through different mechanisms:

- Aerobic exercise: Activities such as walking, running, cycling, and swimming increase mitochondrial density and metabolic flexibility, allowing muscle cells to utilize glucose and fatty acids more efficiently for energy production. Regular aerobic activity has been shown to enhance insulin signaling pathways, decrease hepatic glucose output, and reduce postprandial blood sugar spikes [54].
- Resistance training: Strength-based exercises, including weightlifting, resistance bands, and bodyweight exercises, increase skeletal muscle mass, which is a primary site for glucose disposal. More muscle mass translates to greater glucose storage capacity, improved resting metabolic rate, and enhanced insulin sensitivity, even at rest. Resistance training has also been shown to lower fasting blood glucose levels and reduce glycated hemoglobin (HbA1c), a marker of long-term blood sugar control [62].

Also, exercise reduces chronic low-grade inflammation, a major contributor to insulin resistance [63]. Physical activity lowers levels of pro-inflammatory cytokines such as tumor necrosis factor-alpha (TNF-α) and interleukin-6 (IL-6) while increasing levels of interleukin-10 (IL-10), an anti-inflammatory marker. By reducing inflammation, exercise helps preserve pancreatic function, improve vascular health, and optimize metabolic efficiency [63,64].

### 4.2. Cardiovascular Benefits and Lipid Profile Improvement

Dyslipidemia, a hallmark of metabolic syndrome, significantly increases the risk of cardiovascular disease by promoting atherosclerosis, hypertension, and vascular dysfunction [35]. Individuals with metabolic syndrome often present with elevated triglycerides, increased LDL cholesterol, and reduced HDL cholesterol. All of this can contribute to arterial plaque formation, restricted blood flow, and increased cardiac strain. Regular physical activity is essential for regulating lipid metabolism and improving vascular function. It also helps reduce cardiovascular risk factors [55].

Exercise has been shown to lower triglyceride levels, reduce LDL cholesterol, and increase HDL cholesterol, leading to improved cholesterol balance and reduced arterial plaque accumulation [65]. Aerobic exercise, such as walking, jogging, cycling, and swimming, is particularly effective in enhancing lipid metabolism. During aerobic activity, the body increases fat oxidation, which helps reduce triglyceride storage and lower LDL cholesterol. In addition, aerobic exercise upregulates lipoprotein lipase (LPL), an enzyme responsible for breaking down triglycerides, allowing them to be used as an energy source rather than accumulating in the bloodstream [66]. Exercise also enhances reverse cholesterol transport, a process in which HDL cholesterol removes excess cholesterol from circulation and transports it to the liver for excretion, reducing the risk of arterial blockages [66,67].

Resistance training further supports cardiovascular health by increasing lean muscle mass which, in turn, boosts resting metabolic rate and promotes lipid utilization. More muscle mass improves glucose and fat metabolism, reducing the likelihood of fat being stored in the bloodstream as LDL cholesterol and triglycerides. Research has shown that individuals who engage in resistance training experience greater reductions in LDL cholesterol and triglycerides while simultaneously increasing HDL cholesterol, leading to a more favorable lipid profile [68]. Strength training also improves vascular elasticity, which helps lower blood pressure and enhances circulatory efficiency [68,69].

The combination of aerobic and resistance training provides synergistic effects in improving lipid metabolism, reducing fat accumulation, and optimizing cardiovascular function. Aerobic exercise is particularly beneficial for cholesterol clearance and triglyceride reduction, while resistance training supports muscle retention, metabolic efficiency, and long-term lipid balance. Together, these exercise modalities offer comprehensive cardiovascular protection, reducing the overall burden of dyslipidemia and CVD risk in individuals with metabolic syndrome [67]. Regular physical activity not only improves lipid profiles, but also promotes better blood circulation, heart function, and vascular health, making it a critical component of metabolic syndrome management [70].

### 4.3. Reduction of Inflammation and Oxidative Stress

Regular physical activity is a powerful tool for reducing systemic inflammation and oxidative stress, both of which are key drivers of metabolic syndrome, insulin resistance, and cardiovascular disease [71]. Chronic low-grade inflammation is a defining characteristic of metabolic syndrome, contributing to dysfunctional insulin signaling, endothelial damage, and lipid imbalances [72]. Exercise plays a critical role in modulating the inflammatory response by decreasing pro-inflammatory cytokines, such as IL-6 and TNF-α, while simultaneously increasing anti-inflammatory markers like IL-10 [71,72,73]. This shift in the body’s inflammatory profile helps improve insulin sensitivity, vascular health, and metabolic efficiency.

Beyond its effects on inflammation, exercise also combats oxidative stress, a condition in which excessive free radicals overwhelm the body’s natural antioxidant defenses, leading to cellular damage and metabolic dysfunction [74]. Physical activity enhances the production of endogenous antioxidant enzymes, such as superoxide dismutase and glutathione peroxidase, which help neutralize reactive oxygen species and reduce oxidative damage. Exercise also improves mitochondrial function, increasing the efficiency of energy production and reducing metabolic strain, further lowering the risk of chronic disease progression [74,75].

### 4.4. Weight Management and Fat Distribution

Excess visceral fat, the deep abdominal fat surrounding internal organs, is one of the most dangerous contributors to metabolic syndrome. It actively promotes insulin resistance, chronic inflammation, and cardiovascular dysfunction [76]. Unlike subcutaneous fat, which sits just beneath the skin, visceral fat is metabolically active. It releases pro-inflammatory cytokines and disrupts hormonal balance, leading to impaired glucose metabolism and increased lipid accumulation. Because of its strong link to metabolic dysfunction, reducing visceral fat is a key target for managing metabolic syndrome [48].

Regular exercise plays a critical role in reducing total body fat but, more importantly, it is particularly effective in targeting visceral adiposity. Aerobic exercise, such as brisk walking, running, cycling, and swimming, has been shown to increase fat oxidation and caloric expenditure, leading to significant reductions in central adiposity [66]. This form of exercise helps shift the body’s energy balance toward burning stored fat, particularly in the abdominal region, where excess fat accumulation poses the greatest health risks [77].

Resistance training further enhances body composition improvements by building lean muscle mass, which in turn boosts resting metabolic rate and enhances glucose utilization. More muscle tissue increases the body’s ability to store and process glucose effectively, reducing the likelihood of excess blood sugar being converted into fat [68]. Research has shown that combining aerobic and resistance training leads to greater reductions in visceral fat and improved insulin sensitivity. This combination also enhances metabolic flexibility, allowing individuals to better regulate energy balance and prevent weight regain [62,78].

### 4.5. The Role of High-Intensity Interval Training (HIIT)

HIIT has emerged as an efficient and highly effective exercise strategy for individuals with metabolic syndrome. Unlike traditional steady-state exercise, HIIT consists of short bursts of intense effort followed by brief recovery periods, allowing individuals to achieve substantial metabolic and cardiovascular benefits in a shorter amount of time [57]. This training method has gained attention for its ability to rapidly improve insulin sensitivity, enhance fat metabolism, and optimize cardiovascular function, making it particularly useful for those managing metabolic dysfunction [79].

One of the key benefits of HIIT is its ability to enhance glucose uptake in muscle cells by stimulating GLUT4 translocation, a process that enables skeletal muscle to absorb glucose more efficiently, even in the absence of insulin [80]. This mechanism is particularly beneficial for individuals with insulin resistance, as it helps reduce circulating blood sugar levels and alleviates the demand on pancreatic beta cells. Notably, HIIT has been shown to improve mitochondrial function, increasing the body’s ability to generate energy more efficiently and reduce oxidative stress, both of which contribute to better metabolic health [80,81].

Compared to moderate-intensity continuous exercise, HIIT has demonstrated superior fat-burning capacity and greater reductions in visceral fat, which is strongly associated with cardiovascular disease, insulin resistance, and systemic inflammation [82]. The high-intensity intervals stimulate a greater post-exercise oxygen consumption effect, meaning the body continues burning calories at an elevated rate long after the workout has ended. This prolonged metabolic boost enhances lipid metabolism, leading to lower triglyceride levels and improved cholesterol profiles. It also helps reduce central adiposity, which is a critical factor in managing metabolic syndrome [82,83].

## 5. Synergistic Effects of Plant-Based Nutrition and Exercise

While plant-based nutrition and exercise are individually powerful interventions for managing metabolic syndrome, their combined effects may lead to even greater improvements in metabolic health. Both play complementary roles in enhancing glucose metabolism, reducing inflammation, optimizing body composition, and improving cardiovascular function [49]. Together, they create a holistic and sustainable strategy for preventing and managing metabolic syndrome more effectively than either intervention alone (Table 4).

A plant-based diet provides essential nutrients, fiber, and bioactive compounds that contribute to improved insulin sensitivity, healthier lipid profiles, and reduced inflammation. The high fiber content slows glucose absorption, promoting stable blood sugar levels and reducing insulin resistance [84]. Antioxidants and polyphenols from plant foods help lower oxidative stress and systemic inflammation, further supporting metabolic function [44]. Regular physical activity enhances these benefits by increasing metabolic efficiency, promoting muscle glucose uptake, and stimulating fat oxidation [78]. Exercise helps regulate energy balance, preventing excess fat accumulation, particularly visceral fat, which is strongly linked to insulin resistance and cardiovascular disease [85]. Also, resistance training contributes to muscle mass retention, which improves long-term glucose disposal and metabolic rate [84,85,86].

### 5.1. Enhanced Insulin Sensitivity and Glucose Regulation

Both plant-based diets and exercise independently enhance insulin sensitivity, but when combined, they produce additive metabolic benefits that result in more effective blood sugar regulation [15]. This approach targets multiple mechanisms involved in glucose metabolism, insulin function, and long-term metabolic stability, making it a highly effective strategy for reducing the risk of type 2 diabetes and improving overall metabolic health [15,29].

A fiber-rich plant-based diet plays a crucial role in slowing glucose absorption, which helps maintain steady blood sugar levels and prevents postprandial glucose spikes [87]. The soluble fiber found in whole grains, legumes, fruits, and vegetables forms a gel-like substance in the digestive tract, delaying carbohydrate digestion and absorption [88]. This process reduces glycemic fluctuations, decreasing the insulin demand on the pancreas and improving long-term insulin sensitivity [26]. To add, plant-based diets are rich in polyphenols and anti-inflammatory compounds, which help combat oxidative stress and chronic inflammation, both of which contribute to insulin resistance [87,88,89].

Exercise further enhances glucose regulation by stimulating GLUT4 translocation, a process that allows muscle cells to absorb glucose without the need for insulin [90]. This insulin-independent mechanism is particularly beneficial for individuals with insulin resistance, as it provides an alternative pathway for lowering blood sugar levels and reducing the pancreas’s workload [85]. Equally, regular physical activity improves mitochondrial function, increasing the body’s ability to burn glucose and fatty acids for energy, reducing the risk of glucose accumulation and metabolic dysfunction [60].

### 5.2. Greater Cardiovascular Protection

Cardiovascular health is a primary concern for individuals with metabolic syndrome, as the condition significantly increases the risk of atherosclerosis, hypertension, and cardiovascular disease [1]. Both plant-based diets and regular exercise play vital roles in improving lipid profiles, enhancing vascular function, and supporting overall heart health [91]. When combined, these lifestyle interventions create a synergistic effect, offering greater cardiovascular protection than either strategy alone [1,14,91].

A plant-based diet contributes to heart health by reducing key risk factors such as high LDL cholesterol, elevated triglycerides, and systemic inflammation [92]. Diets rich in unsaturated fats from nuts, seeds, and olive oil help lower LDL cholesterol, the type associated with plaque buildup in the arteries, while increasing HDL cholesterol, which plays a protective role by aiding in cholesterol clearance from the bloodstream [20]. Equally, the soluble fiber found in whole grains, legumes, and vegetables binds to cholesterol in the gut, promoting its excretion and further lowering circulating LDL levels [39]. The antioxidants and polyphenols in plant-based foods help reduce oxidative stress, protecting the endothelial lining of blood vessels and improving vascular function [92,93].

Regular physical activity enhances these cardiovascular benefits by optimizing lipid metabolism, improving circulation, and reducing arterial stiffness. Aerobic exercise, such as brisk walking, cycling, or swimming, increases LPL activity, an enzyme that helps break down triglycerides and clear them from the blood [94]. Resistance training further aids in lipid regulation by increasing lean muscle mass, which improves glucose and fat metabolism while supporting long-term cardiovascular efficiency [55]. Exercise also stimulates the production of nitric oxide, a molecule that helps dilate blood vessels, improve endothelial function, and lower blood pressure, reducing strain on the cardiovascular system [94,95].

To add, plant-based diets are naturally lower in sodium and higher in potassium, a combination that supports healthy blood pressure regulation by counteracting the effects of excess sodium on fluid balance [96]. When combined with regular exercise, which supports vascular elasticity and circulation, this approach offers a natural way to reduce hypertension. Together, they create a comprehensive strategy for lowering cardiovascular disease risk [97].

### 5.3. Amplified Anti-Inflammatory Effects

Chronic low-grade inflammation is a major contributor to metabolic syndrome, driving insulin resistance, endothelial dysfunction, and cardiovascular disease. Persistent inflammation disrupts normal metabolic processes, leading to dysregulated blood sugar levels, lipid imbalances, and increased oxidative stress [72]. A combination of plant-based nutrition and regular exercise provides a powerful anti-inflammatory strategy, reducing systemic inflammation and oxidative damage while improving overall metabolic health [98].

A plant-based diet is rich in anti-inflammatory compounds, including polyphenols, flavonoids, and omega-3 fatty acids from sources such as berries, leafy greens, nuts, seeds, and flaxseeds [6]. These bioactive compounds help suppress pro-inflammatory pathways, neutralize free radicals, and lower oxidative stress [19]. Polyphenols and flavonoids inhibit the production of inflammatory cytokines while promoting cellular repair and immune regulation. Also, the fiber content in plant-based diets supports gut microbiome diversity, which plays a key role in regulating immune function and reducing systemic inflammation [99]. A well-balanced gut microbiome produces SCFAs that help reduce inflammation and maintain metabolic stability [45].

Exercise further amplifies these anti-inflammatory effects by lowering levels of pro-inflammatory cytokines such as TNF-α and IL-6. At the same time, it increases IL-10, a key anti-inflammatory marker [100]. These changes help mitigate chronic inflammation, reducing vascular damage, insulin resistance, and metabolic dysfunction. Regular physical activity also enhances mitochondrial function and increases antioxidant enzyme activity, improving cellular resilience against oxidative stress. It strengthens the body’s natural antioxidant defenses, helping to protect tissues from damage. As a result, exercise plays a key role in reducing the impact of chronic inflammation [74,75].

### 5.4. Optimized Weight Management and Fat Reduction

Achieving and maintaining a healthy weight is a critical component of both preventing and managing metabolic syndrome [101]. Excess body fat, particularly visceral fat, is strongly linked to insulin resistance, chronic inflammation, and cardiovascular disease. Sustainable weight management is essential for improving metabolic health [102]. A combination of plant-based nutrition and regular exercise provides a highly effective, long-term strategy for fat reduction, appetite regulation, and improved body composition [101,102,103].

A plant-based diet naturally supports satiety and appetite control due to its high fiber and water content, which promotes fullness while lowering caloric intake without the need for restrictive dieting [84]. Dietary fiber slows digestion, reducing postprandial glucose spikes and enhancing the release of satiety hormones, such as GLP-1 and PYY. These hormones signal fullness to the brain, helping individuals naturally regulate hunger and caloric consumption while preventing overeating and cravings [50]. Fiber also stabilizes blood sugar levels, reducing insulin fluctuations. This helps prevent the increased fat storage often associated with these fluctuations [22].

Exercise complements these dietary effects by increasing energy expenditure, enhancing fat oxidation, and promoting lean muscle mass development. Aerobic exercise, including activities such as brisk walking, running, cycling, and swimming, helps mobilize and burn stored fat, with a particular impact on visceral fat reduction [66]. Resistance training, on the other hand, plays a crucial role in preserving and building muscle mass during weight loss, which is essential for maintaining a higher resting metabolic rate. More muscle mass improves the body’s ability to efficiently utilize glucose and fatty acids, reducing the likelihood of excess fat accumulation [68].

Visceral fat is a key driver of insulin resistance, systemic inflammation, and cardiovascular dysfunction. Targeting it through a combination of plant-based nutrition and physical activity offers an effective approach to long-term weight management. This strategy also supports overall metabolic health improvement [104]. This dual approach not only supports healthy weight loss, but also ensures that the body remains metabolically efficient, preventing weight regain and reducing the risk of metabolic complications over time [104,105].

### 5.5. Long-Term Adherence and Lifestyle Sustainability

Sustained adherence to healthy eating patterns and regular physical activity is essential for the long-term management of metabolic syndrome [106]. Many individuals struggle with maintaining lifestyle changes. However, adopting a plant-based diet and engaging in regular exercise can reinforce one another, creating a self-sustaining cycle of health-promoting behaviors [9]. This interconnected relationship makes it easier to maintain weight loss, metabolic improvements, and cardiovascular benefits over time (Table 5) [50].

Research suggests that individuals who exercise regularly are more likely to adopt healthier dietary habits, such as increased consumption of fruits, vegetables, and whole foods. Physical activity has been shown to influence food preferences, leading to a greater inclination toward nutrient-dense foods while reducing cravings for processed and high-fat foods [107]. Those following a plant-based diet often experience higher energy levels, improved recovery, and enhanced physical performance, making them more likely to engage in consistent physical activity. This positive cycle creates a strong connection between diet and exercise. As a result, it fosters long-term adherence to both habits and supports a sustainable approach to metabolic health [107,108].

Another key factor in lifestyle sustainability is the psychological and behavioral impact of small, consistent changes. Gradual improvements in diet and exercise habits create positive reinforcement loops, helping individuals feel better and stay motivated [109]. As people experience these benefits, they are more likely to maintain their health journey. Social support and access to healthy food options are crucial in long-term success. In addition, finding enjoyable physical activities increases adherence and makes sustainable health habits more achievable [109,110].

## 6. Discussion

This narrative review examined the impact of plant-based nutrition and exercise, both individually and in combination, on metabolic syndrome. We focused on how these lifestyle factors influence insulin sensitivity, cholesterol levels, inflammation, body composition, and heart health. What makes this review unique is that it combines research on both diet and exercise, a combination that has not been explored as often. Most existing reviews focus on just one area. Marrone et al. (2021) reviewed the metabolic benefits of vegan diets, demonstrating improvements in blood glucose control, lipid profiles, and inflammation through a plant-based diet [9]. Bird and Hawley (2017) examined how physical activity, particularly aerobic exercise, enhances insulin sensitivity and supports metabolic function [53]. In contrast, our review explores the combined and potentially synergistic effects of both strategies. Unlike previous reviews that have independently examined the effects of plant-based diets or physical activity, this review provides a comprehensive synthesis of both. By exploring shared metabolic pathways, such as insulin sensitivity, lipid metabolism, inflammation, and body composition, this review contributes a novel, integrative perspective to the management of metabolic syndrome. By undertaking this, we aim to provide a more complete and practical understanding of how integrated lifestyle changes can help manage metabolic syndrome more effectively.

Our analysis of 114 peer-reviewed articles highlights strong evidence supporting both interventions as effective strategies for metabolic health improvement. Plant-based diets, particularly whole-food, Mediterranean, and flexitarian approaches, significantly reduce insulin resistance, dyslipidemia, and chronic inflammation [21]. Similarly, structured exercise programs, including aerobic, resistance, and HIIT, enhance glucose metabolism, improve lipid regulation, and reduce visceral fat accumulation [80].

The synergy between these two interventions arises from complementary physiological effects. Plant-based diets improve insulin signaling through high intake of soluble fiber and polyphenols, which reduce postprandial glucose spikes and systemic inflammation [29,32,89]. Exercise enhances insulin-independent glucose uptake through GLUT4 translocation, improves mitochondrial function, and increases resting metabolic rate [60,61,62]. These mechanisms intersect to lower insulin demand and improve glycemic control more effectively when combined.

Despite the well-established benefits of both plant-based diets and exercise, their combined effects remain underexplored. The available literature suggests that these interventions work synergistically. Plant-based diets provide essential nutrients that support muscle function, recovery, and energy metabolism, while exercise amplifies the metabolic benefits of dietary changes [106,111]. However, heterogeneity in definitions of “plant-based” eating patterns, ranging from vegan to semi-vegetarian or Mediterranean, creates variability in outcomes [11]. Similarly, exercise interventions differ in terms of intensity, frequency, and modality, which complicates cross-study comparisons [54,67]. Additionally, most clinical trials are short-term, and lack follow-up data beyond 12 months, which limits conclusions about sustainability. For example, a 16-week randomized controlled trial by Barnard et al. demonstrated significant weight loss with a low-fat vegan diet; however, it provided no follow-up data beyond the intervention period [91].

There are also limitations in population diversity, as many studies focus on relatively healthy or affluent participants, which reduces their generalizability to underserved groups. In the Adventist Health Study-2, for instance, over 90% of participants identified as non-Hispanic white and were college-educated, which limits applicability to more diverse populations [52]. Cultural food preferences, economic access, and healthcare disparities must be taken into account when applying lifestyle recommendations to diverse communities [112,113]. Long-term adherence remains a critical barrier to success, with factors such as accessibility, cultural preferences, and behavioral motivation influencing compliance. A recent umbrella review of over 40 studies found that lack of motivation, time constraints, and social support were among the most common barriers to adherence in lifestyle interventions for chronic disease [114]. Additionally, as a narrative review, our study did not follow systematic review protocols or include a formal risk-of-bias assessment. Future research should include long-term, randomized controlled trials with diverse populations to assess the effectiveness and sustainability of combined diet and exercise interventions more accurately.

## 7. Conclusions

Metabolic syndrome is a significant public health concern that increases the risk of cardiovascular disease, type 2 diabetes, and other chronic conditions. Plant-based nutrition and regular exercise are among the most effective non-pharmacological strategies for prevention and management, as they improve insulin sensitivity, reduce inflammation, and enhance cardiovascular and metabolic function. While strong evidence supports their individual and combined benefits, further research is needed to refine dietary and exercise recommendations, improve long-term adherence, and assess their effectiveness across diverse populations. Integrating these lifestyle strategies into public health initiatives and clinical practice is essential to making them accessible and sustainable. By prioritizing plant-based nutrition and physical activity, individuals can take proactive steps to improve metabolic health, reduce chronic disease risk, and enhance overall well-being. However, it is also important to recognize that poorly planned vegan diets may lead to deficiencies in key nutrients such as vitamin B12, iron, omega-3 fatty acids, and complete protein. These risks highlight the need for informed dietary planning, especially in clinical practice, to ensure both efficacy and safety in long-term metabolic health strategies.

## Figures and Tables

**Table 1:** Comparison of plant-based dietary patterns and their metabolic benefits (Up arrows mean increase and down arrows mean decrease).

Dietary Pattern	Macronutrient Composition	Effects on Insulin Sensitivity	Effects on Lipid Profiles	Effects on Inflammation
Vegan	High carb, moderate protein, low fat	Strong improvement due to high fiber, low glycemic load [5,6,14,15]	↓ LDL, ↑ HDL, ↓ total cholesterol [5,6,14]	↓ CRP, ↑ antioxidant capacity [6,9,14]
Vegetarian	Moderate carb, moderate protein, moderate fat	Moderate improvement due to some animal product inclusion [5,7]	↓ LDL, moderate effect on HDL [5,7,16,17]	Moderate reduction in inflammatory markers [7,14]
Mediterranean (Plant-Focused)	Moderate carb, high unsaturated fat	Significant improvement due to polyphenols and unsaturated fats [10,14,18]	↓ LDL, ↑ HDL, ↓ triglycerides [16,17,18]	Strong anti-inflammatory effects [10,14,19]
Flexitarian	Balanced macronutrient intake	Moderate improvement due to flexible plant intake [5,12]	Moderate reduction in LDL, slight increase in HDL [5,13,20]	Mild to moderate anti-inflammatory benefits [5,12,21]
Whole-Food Plant-Based	High fiber, low processed food	Strongest improvement due to high fiber and nutrient density [5,6,14,21]	Strongest reduction in LDL and triglycerides [14,21]	Strongest reduction in systemic inflammation [14,21]

**Table 2:** Nutrient profiles and calorie density of common plant-based foods relevant to metabolic health.

Food	Primary Nutrients	Metabolic Benefits	Calorie Density (kcal/g)
Kale	Magnesium, vitamin K, polyphenols	Supports blood pressure, reduces inflammation, improves endothelial function	50
Lentils	Plant-based protein, fiber, iron	Improves satiety, stabilizes glucose, supports muscle maintenance	120
Avocado	Monounsaturated fats, potassium, fiber	Improves lipid profiles, enhances satiety, regulates blood pressure	160
Oats	Soluble fiber (beta-glucan), manganese	Lowers LDL cholesterol, improves glycemic control	370
Blueberries	Polyphenols (anthocyanins), vitamin C	Antioxidant effects, reduces oxidative stress and inflammation	60
Tofu	Plant-based protein, isoflavones, calcium	Aids in muscle maintenance, supports cardiovascular health	150
Chia Seeds	Omega-3 fatty acids, fiber, antioxidants	Reduces triglycerides, improves insulin sensitivity	480
Sweet Potatoes	Beta-carotene, vitamin C, fiber	Supports immune function, regulates glucose, promotes gut health	90
Walnuts	Omega-3 fatty acids, antioxidants, protein	Reduces inflammation, improves lipid profile	660
Black Beans	Fiber, protein, folate, iron	Enhances glycemic control, promotes satiety, improves gut microbiota	140

**Table 3:** Effects of different exercise modalities on metabolic syndrome components (Up arrows mean increase and down arrows mean decrease).

Exercise Type	Impact on Insulin Sensitivity	Impact on Lipid Profile	Impact on Body Composition	Impact on Inflammation
Aerobic (Running, Cycling)	↑ GLUT4 translocation, ↑ glucose uptake [53,55]	↓ LDL, ↓ triglycerides, ↑ HDL [55]	↓ Visceral fat, improved BMI [53,54]	↓ CRP, IL-6, improved endothelial function [53,54]
Resistance Training	↑ Muscle glucose uptake, ↑ insulin efficiency [56]	↑ HDL, ↓ LDL, ↓ triglycerides [55,56]	↑ Lean muscle mass, ↑ resting metabolic rate [55,56]	↓ TNF-α, ↑ IL-10, reduced inflammation [55,56]
HIIT	↑ Mitochondrial function, ↑ metabolic flexibility [57]	Rapid fat oxidation, ↑ HDL [55,56,57]	Rapid fat loss, esp. visceral fat [57]	Strong anti-inflammatory response [57]
Functional Training	↑ Insulin sensitivity via dynamic movement [55]	Moderate lipid improvements [53,54,55]	↑ Strength, ↓ fat mass, improved mobility [53,54]	Moderate anti-inflammatory effects [53,57]

**Table 4:** Combined benefits of plant-based diet and exercise on metabolic syndrome (Up arrows mean increase and down arrows mean decrease).

Combined Effect	Mechanism	Supporting Reference(s)
Enhanced insulin sensitivity	↑ GLUT4 translocation, ↓ insulin resistance, ↑ fiber and polyphenols	[15,26,29]
Improved lipid metabolism	↓ LDL and triglycerides, ↑ HDL, ↓ saturated fat intake	[16,17,20,37,67]
Reduced systemic inflammation	↓ TNF-α, IL-6; ↑ IL-10; ↑ antioxidants and SCFA production	[19,43,45,64]
Lower visceral fat	↑ fat oxidation, ↓ glycemic spikes, ↑ satiety, ↑ lean muscle mass	[52,68,70,78]

**Table 5:** Behavioral and adherence benefits of combined interventions.

Factor	Description	Supporting Reference(s)
Mutual reinforcement	Diet and exercise promote each other’s success	[9,50,107]
Improved food choices	Exercise increases preference for whole foods	[107,108]
Enhanced energy and mood	Plant-based diets and regular activity support psychological well-being	[108,109]
Social support and accessibility	Key to sustaining long-term behavior changes	[110,111,112,113,114]
Habit formation and motivation	Small consistent changes reinforce health behaviors	[109,110]

## Data Availability

No new data were created or analyzed in this study. Data sharing is not applicable to this article as it is a narrative review of existing literature.
